# Differences in ocular vessels support a distinct vasculopathy in parietal cortical atrophy

**DOI:** 10.1002/alz70856_101744

**Published:** 2025-12-24

**Authors:** William Robert Kwapong, Liyong Wu, Min Chu

**Affiliations:** ^1^ Xuanwu Hospital, Capital Medical University, Beijing, Beijing, China; ^2^ Beijing Key Laboratory of Geriatric Cognitive Disorders and Neurodegenerative, Beijing, China; ^3^ Xuanwu Hospital, Capital Medical University, Beijing, China

## Abstract

**Background:**

Alzheimer's disease (AD) encompasses a variety of typical and atypical phenotypes but the vascular changes between these phenotypes are unclear. Posterior cortical atrophy (PCA) is a type of AD that frequently displays visual dysfunction and overlaps with typical Alzheimer's disease (tAD). Retinal vessels share ontogeny, size and physiology features with cerebral microcirculation and retinal vascular changes is associated with AD. We compared the ocular vascular changes as a surrogate for cerebral microcirculation in patients with PCA and tAD.

**Method:**

We prospectively recruited AD patients who were Aß positive and Aß negative cognitively unimpaired (CU) controls. All participants underwent neuropsychological assessment, neuroimaging and optical coherence tomography angiography (OCTA) imaging. Optic nerve head (ONH) and macula (superficial vascular complex, SVC and inner retinal vascular complex, IVC) were performed with the OCTA (Zeiss 5000). An automated algorithm was used to quantify vessel tortuosity and branching complexity of ocular vessels from OCT angiograms.

**Result:**

Sixteen PCA, 30 tAD and 34 controls were included. AD showed significantly increased vascular tortuosity and less complex branching in the ONH and macula compared to CU (*p* < 0.001). Similarly, AD showed increased vascular tortuosity and less complex branching in the ONH and macula compared to CU (*p* < 0.05). PCA showed increased vascular tortuosity (*p* = 0.024) and less complex branching (*p* = 0.004) in the ONH and macula when compared to tAD. We also showed significant correlation between ONH vascular changes and medial temporal lobe atrophy scores in PCA and tAD respectively (all *p* < 0.05).

**Conclusion:**

Ocular microvasculature is sparser and his more tortuous in PCA than tAD. These findings suggest that ocular imaging has the potential to be used to detect microvascular changes in PCA and tAD.

